# Pneumatosis Intestinalis in Adult Bilateral Lung Transplant Patients: A Single Institution Experience and Literature Review

**DOI:** 10.1155/2020/5023948

**Published:** 2020-07-13

**Authors:** Eric Christiansen, Nisha Singh, Amy Trahan, Sofya Tokman, David Row, Olga Kalinkin

**Affiliations:** ^1^Department of Radiology, St. Joseph's Hospital and Medical Center, Phoenix, AZ, USA; ^2^Department of Medicine, St. Joseph's Hospital and Medical Center, Phoenix, AZ, USA; ^3^Norton Thoracic Institute, St. Joseph's Hospital and Medical Center, Phoenix, AZ, USA; ^4^Department of Surgery, St. Joseph's Hospital and Medical Center, Phoenix, AZ, USA

## Abstract

Pneumatosis intestinalis (PI) is a radiologic finding which is characterized by the accumulation of gas within the bowel wall. This radiologic finding is traditionally thought of in the sense of intestinal ischemia. An uncommon cause of this finding is post organ transplantation. We did an institutional and literature review of this finding to demonstrate its distinct imaging features and benign nature. It was observed to occur in approximately 5.2% of patients post lung transplant (23/442). On imaging, it displays an expansile/bubbly appearance of gas within the bowel wall that is distinct from the traditional findings seen in intestinal ischemia. Clinical review showed that posttransplant patients with PI can be successfully managed conservatively with early enteral nutrition, oxygen, antibiotics, and limited follow-up imaging. With the increasing use of organ transplantation, PI is being diagnosed with increased frequency. It is important to let clinicians know of this entity and its potential outcomes.

## 1. Introduction

Pneumatosis intestinalis (PI) is a radiologic and pathologic finding which is defined as accumulation of gas within the submucosa or subserosa of the small or large bowel [1]. PI was first reported by Du Vernoi in 1730 during cadaveric dissection [2]. It is a clinical condition with a wide spectrum of etiologies and outcomes, ranging from completely benign to life threatening. PI is most commonly thought of in critical scenarios such as intestinal ischemia, necrotizing enterocolitis, mesenteric vascular disease, and intestinal obstruction [3]. Benign PI is a less common entity, usually found incidentally on plain radiograph or CT and is typically seen in conditions related to chronic immunosuppression such as solid organ transplant and graft vs. host disease (GVHD), as well as chronic obstructive pulmonary disease (COPD), asthma, scleroderma, or as a complication of endoscopy [4].

Pneumoperitoneum, mesenteric gas, and portal venous gas are additional imaging findings often seen in the setting of PI that have not been proven to correlate with severity of disease [5]. Pneumatosis intestinalis associated with solid organ transplantation poses a unique challenge since these patients have recently undergone an extensive operation and are on high-dose immunosuppression; thus, they may be poor surgical candidates. There have been a number of case reports of benign PI in the setting of lung transplant over the years, including two more recent small case series, Thompson et al. with 7 patients and Chandola et al. with 10 patients, which demonstrated this to be a benign entity that can be managed conservatively [5, 6].

## 2. Methodology

A thorough departmental review was performed using Montage, which was queried for patients who underwent lung transplant and subsequently developed pneumatosis intestinalis. Between March 2015 and June 2019, there were 442 lung transplants performed with 23 patients also having PI diagnosed on CT. These patients had CT scans to evaluate the extent of pneumatosis after it was incidentally found on a plain chest radiograph. Each of the scans was individually analyzed by a radiologist for additional findings and characterization of the PI. Systemic review of clinical notes and electronic medical records was performed in order to collect information on patient demographics, clinical course, hospital stay, laboratory values, medications, and any interventions.

## 3. Results

From March 2015 to June 2019, 442 lung transplants were performed in which 23 adults were observed to incidentally develop PI (5.2%, Table 1). There were 13 men and 9 women with mean age of 62 years (range, 37-79 y). The primary diagnosis and reason for lung transplant was pulmonary fibrosis (*n* = 16), scleroderma-related interstitial lung disease (*n* = 4), sarcoidosis (*n* = 2) and alpha-1 antitrypsin-related COPD (*n* = 1). Mean time to development of PI post lung transplant was 168 days, range 5 to 1477 days, and median of 47 days. Placement of gastrojejunostomy (GJ) tube after transplant and before development of PI was seen in 19 patients (83%). The mean time from placement of the GJ tube to development of PI was 66 days, range 3 to 519 days, and median of 37 days.

Distribution of PI was observed with involvement of the cecum in 78% (*n* = 18), ascending colon in 87% (*n* = 20), transverse colon in 57% (*n* = 13), and descending colon in 26% (*n* = 6). Pneumoperitoneum was seen in 65% (*n* = 15) of cases, and mesenteric gas in 57% (*n* = 13). Portal venous gas was only seen in 2 cases (7%).

The majority of lung transplant recipients with PI were managed conservatively with a combination of bowel rest, supplemental oxygen, and antibiotics. Patients remained nil per os (NPO) for an average of 1.1 days after diagnosis (range, 0-4). Diet was typically advanced by starting out with a clear liquid diet or tube feeds depending on the patient, which progressed daily as symptoms improved until their goal nutrition was achieved. Management varied based on admitting provider and was noted to become more standardized in recent years. Of the 21 subjects who were treated as inpatients, 100% were treated with high flow oxygen typically at 15 L, 52% were administered Flagyl, and 2 patients (9%) ended up requiring surgery. Almost all patients were on antibiotic coverage; however, we specifically looked at Flagyl due to its documented use in the literature in these patients. Improvement of PI is typically assessed radiographically with either an abdominal X-ray or an abdominal CT scan. The colorectal surgery (CRS) service is typically consulted for GI-related issues with all transplant patients. The average length of CRS involvement in patients with PI is 3.5 days (range, 0-16); they typically stay involved with patient care until GI symptoms have resolved and the patient is tolerating a diet. Follow-up imaging was also assessed; of the 23 patients, 19 patients received follow-up radiographs (83%), while 3 patients received follow-up CT scans (13%). On average, patients had 3.5 abdominal X-rays (range, 0-9) and 0.2 abdominal CTs (range, 0-2).

Pneumatosis intestinalis was asymptomatic in the majority of patients (12, 57%). Eleven patients complained of gastrointestinal symptoms with 10 reporting nausea and diarrhea and only 2 reporting abdominal pain. Notably, diarrhea and nausea were long standing in many of these patients and likely preceded the development of PI.

There are several laboratory values that can help predict prognosis in patients with PI [3]. These include a lactic acid level > 2.0 (mmol/L), bicarbonate > 20 (mEq/L), amylase > 200 (U/L), and pH level < 7.3 [3]. In this cohort, the mean lactic acid level was 1.3 mmol/L (range, 2.0-3.3) with 5 patients having a level above 2.0 mmol/L. Mean serum bicarbonate was 26.5 mEq/L, and all patients had a serum bicarbonate > 20 mEq/L (range, 22-37). None of the patients had an amylase above 200 U/L with mean serum amylase being 50.6 U/L (range, 17-90). The serum pH was assessed in only 3 patients and ranged from 7.47 to 7.49.

As part of the pretransplant work-up, all patients received a colonoscopy prior to their procedure. Based on the colonoscopy reports, we specifically analyzed the findings involving the ascending and transverse colon looking for any predisposing factors for the development of pneumatosis. Three patients had polyps discovered in the ascending colon; pathology revealed these to be tubular adenomas without high-grade dysplasia. Two patients had diverticula involving the ascending colon without active inflammation, while one patient had active colitis of the ascending colon. All other patients had normal colonoscopy findings.

All lung transplant recipients were immunosuppressed at the time of PI diagnosis, and all were taking prophylactic antimicrobials. The vast majority (22, 96%) were taking a combination of corticosteroids (prednisone), calcineurin inhibitors (tacrolimus), and antiproliferative agents (mycophenolate mofetil). One patient was on 2 types of drug immunosuppression consisting of tacrolimus and prednisone alone. All the patients took valganciclovir for cytomegalovirus prophylaxis, 22 took an azole antifungal for fungal prophylaxis (as coccidioidomycosis is ubiquitous in the southwest of the United States), and 19 took trimethoprim-sulfamethoxazole for pneumocystis pneumonia prophylaxis. There are numerous other antibiotics that only 1 or 2 patients were on, typically for targeting a specific bacterium or due to medication allergies; this list included Ampicillin, Nystatin, Itraconazole, CMV immunoglobulin, Meropenem, Daptomycin, Vancomycin, Micafungin, Piperacillin-Tazobactam, and Atovaquone. All 23 patients tested negative for cytomegalovirus posttransplantation.

## 4. Discussion

Although the mechanism of PI in lung transplant recipients remains uncertain, 3 major theories have been put forth and include the mechanical, the bacterial, and the biochemical [1, 7].The mechanical theory postulates that gas dissects into the bowel wall from increased intraluminal pressure or from increased mediastinal pressure in mechanically ventilated patients via the mediastinum. The bacterial theory suggests that gas-forming bacteria enter the submucosa through mucosal rents and produce gas within the bowel wall, while the biochemical theory suggests that carbohydrates are broken down by bacteria resulting in gas production which is then absorbed into the bowel wall. Notably, there is also a less well-known theory that postulates that PI results from immunosuppression-driven atrophy of the lymphoid tissues within the bowel wall. This atrophy may compromise the integrity of the bowel mucosa and allows gas to dissect into the bowel wall (reference). One additional theory that we wanted to explore was PI forming as a result of feeding tubes; the proposed mechanism being a combination of iatrogenic mucosal injury and increased intraluminal pressures as well as increased bacterial overgrowth in the region of tube feeds [8]. Another common etiology of PI in nontransplant patients is intestinal ischemia; however, due to the nonwatershed distribution and time frame, late postsurgical, seen in our patient population, this does not appear to be a potential etiology.

There are multiple case reports and 2 small case series [5, 6, 9, 10] that have previously been published on PI in the post lung transplant population. Thompson et al. described 6 patients (out of 321, Table 2) with a rate of observation of 2% while Chandola et al. described 10 patients (out of 373, Table 3) with a rate of 2.68% [5, 6]. These are very similar to our rate of observation of 5.2%. However, the true incidence is likely underestimated due to this commonly being an incidental finding in asymptomatic patients. The clinical presentation, radiologic appearance, and clinical outcomes were also similar to ours. Both authors agreed that patients can be managed conservatively in the majority of cases. However, Chandola et al. showed that high serum lactate and portal venous gas were indicative of more severe illness requiring surgical management.

The mean and median to development of PI after transplant were 168 days and 47 days, respectively (range, 5-1477), in our group of patients. Chandola et al. had a mean time of 352.8 days (median 82 days, range 5-2495 days) and Thompson et al. had a mean time of 152 days (median 105 days, range 18-453 days). Many of our subjects (74%) were outpatients at the time of PI diagnosis. The patients that developed PI while still admitted posttransplant typically occurred later in their stay; the extended admissions were all secondary to other issues and not typically a result of the PI. At our institution, it is common for the transplant team to consult the colorectal surgery (CRS) for pneumatosis intestinalis. The surgical team follows the patients until PI improves radiographically and the patient tolerates enteral nutrition. On average, CRS followed the patients for 3.5 days (range, 0-16).

**Figure 1:**
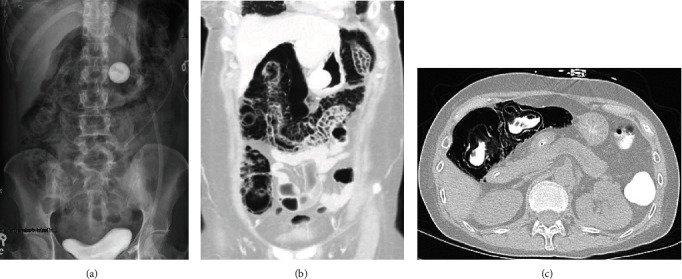
(a) Abdominal radiograph, (b) coronal CT, and (c) axial CT. PI of the ascending and transverse colon with “bubbly/expansile” wall appearance with extensive air cysts in the subserosal and submucosal layers.

**Figure 2:**
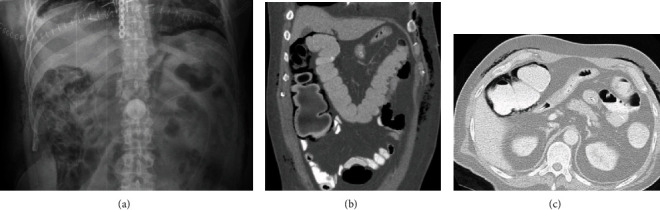
(a) Abdominal radiograph, (b) coronal CT, and (c) axial CT. Pneumatosis intestinalis of the ascending and proximal transverse colon displaying circumferential bubbly/expansile appearance of the colon wall. Notice also the presence of subcutaneous emphysema.

**Figure 3:**
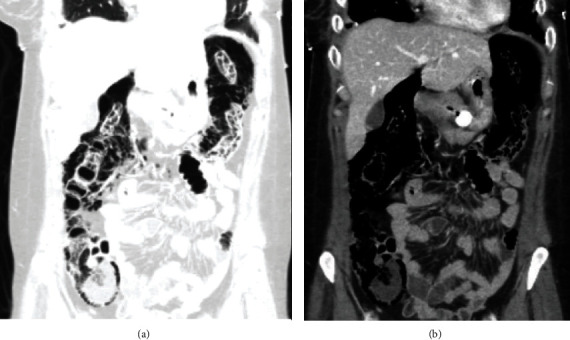
(a) Lung window and (b) soft tissue window. Viewing the bowel through the lung windows allows the reader to better visualize the compartmentalization of the gas, which is located within the colonic wall.

**Figure 4:**
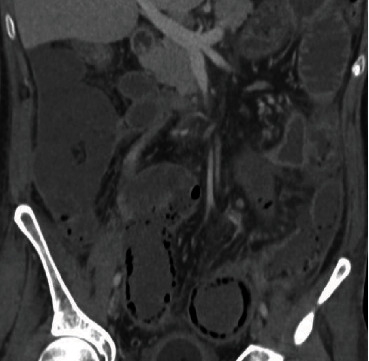
Pneumatosis intestinal in a patient with intestinal ischemia displaying linear small gas bubbles within the colon wall. Note the prominent wall thickening of the colon with the adjacent inflammatory changes of the mesentery.

Imaging findings of pneumatosis intestinalis posttransplant are unique as compared to PI seen in other scenarios such as bowel ischemia. PI in posttransplant patients has a bubble-like/expansile appearance with a significant amount of air within the bowel wall (Figures 1 and 2). In these patients, it is helpful to view the pneumatosis on lung windows in order to properly assess its extent (Figure 3). In contrast, for PI in a patient with intestinal ischemia (Figure 4), imaging findings show more of a thin rim of gas bubbles within the submucosa without the expansile appearance. Although posttransplant PI can appear as a thin layer of gas, it would be unusual for intestinal ischemia to appear as bubble-like/expansile. Thus, if the bubble-like/expansive appearance of PI is seen, it may support a benign etiology and clinical course.

Additional imaging findings that can be seen with PI include pneumoperitoneum, mesenteric gas, and portal venous gas. These findings are not unexpected since the gas within the wall can diffuse/spread into the adjacent mesenteric veins and into the portal venous system. It has been noted in the previous case reports that portal venous gas was associated with increased risk of need for surgery [5, 11]. In our study, neither of the 2 patients that had portal venous gas required surgery or an extended hospital stay.

There were 2 patients that did undergo surgery as a result of the PI in our study. The first patient went to the OR on the day of PI diagnosis which was posttransplant day number 32. This patient had normal labs, including lactic acid, and did not have any abdominal pain. The imaging findings were that of bubble-like/expansile PI of the cecum and ascending colon with mesenteric gas and free air. This was in 2015, and an exploratory laparotomy was performed without any findings. In retrospect, this surgery may have been preventable based on current knowledge. At the time, however, the likely benign clinical course of this entity in the posttransplant patient was not known to the on call surgeon, who was concerned that the patient had ischemic bowel. The second patient that underwent surgery underwent an exploratory laparotomy on day 5 posttransplant due to imaging findings of cecal ischemia and perforation secondary to a closed large bowel obstruction. While this patient was posttransplant and the imaging findings displayed pneumatosis intestinalis, they were not the typical findings of posttransplant PI; it lacked the cystic/bubbly wall. Instead, they were clearly a result of another process, which in this case was a closed loop large bowel obstruction.

Based on our experience, patients with a benign abdominal exam and reassuring laboratory analysis can be managed conservatively with supplemental oxygen, bowel rest, and antibiotics. Our data showed that reinitiation of enteral nutrition early in the patient's admission resulted in decreased hospital stays without adverse effects. The patient's immunosuppressive regimen was not altered during the treatment of PI.

Our report is limited by its relatively small sample size and lack of a control group. However, this is the largest case series to date describing the presentation and management of lung transplant recipients with PI, and our findings support those of smaller case series.

## 5. Conclusion

Pneumatosis intestinalis in lung transplant recipients is a benign entity that presents incidentally. This can be managed conservatively with minimal clinical intervention and follow-up imaging. Management of these patients can be accomplished with early feeding, serial abdominal exams, high flow oxygen, and minimal imaging follow-up. It is likely reasonable to treat this subset of patients outside of the hospital without additional care in the proper clinical setting. Radiologic findings that are suggestive of benign etiology include bubbly/expansile appearance of the colon as well as involvement of the cecum/ascending colon. Free intraperitoneal air is not an indication of severity. Overall our findings align with the 2 previous case series reported in this population. Radiologists and clinicians need to be aware of this entity to prevent unnecessary intervention. Based on analysis of our institutional data, one could presume that the most likely etiology for the development of PI in this patient population is immunosuppressants. In addition, the majority of patients who had instrumentation of the GI tract with a feeding tube also developed PI, and this is likely a contributing factor. It seems that in most clinical scenarios, this is a multifactorial process and subject to further study.

## Figures and Tables

**Table 1:** PI data.

Patient	Age, sex	Primary diagnosis	Time to PI after Tx	Time to PI after GJ tube	Immunosuppression	Symptoms	PI distribution	Pneumoperitoneum	Mesenteric gas	Portal venous gas	Lactic acid	Amylase	Bicarbonate	Follow-up imaging	Management
1	58, M	Sarcoidosis	23	11	P+T+MMF	Abdominal pain, diarrhea	C/A	Y	Y	N	23.5	45	28	XR × 4	NPO × 2 days O_2_ at 15 L Vancomycin, Zosyn, Micafungin
2	43, M	IPF	47	43	P+T+MMF	None	C/A/T/D	N	N	N	1.9		23	XR × 3 CT × 1	NPO × 4 days O_2_ at 15 L Flagyl, Merrem
3	71, F	IPF	53	3	P+T+MMF	N/V/D × 2 days	C/A/T	N	N	N	1.4		29	XR × 9	NPO × 3 days O_2_ at 15 L Flagyl
4	66, F	Sarcoidosis	36	4	P+T+MMF+IVIG	N/V × 3 days	A/T	Y	N	N	1.6	31	23	None	OR for ex lap O_2_ at 15 L (negative) NPO × 1 day
5	67, F	IPF	517	519	P+T+MMF	Diarrhea	A	Y	N	N	0.7	17	25	XR × 3	Flagyl × 14 d O_2_ at 15 L CLD × 1 day
6	66, F	IPF	147		P+T+MMF	Pain × 4 weeks N/V × 4 months	C/A	N	N	N	1.1		23	XR × 6	O_2_ at 15 L NPO × 2 days
7	64, M	IPF	8	10	P+T+MMF	Diarrhea	C/A	N	Y	Y			29	XR × 1	NPO × 1 day O_2_ at 15 L
8	59, M	Scleroderma ILD	5		P+T+MMF	None	C	Y	Y	N			37	XR × 2	OR surgery—cecal ischemia and perforation and closed loop large bowel obstruction
9	69, F	Scleroderma ILD	41	45	P+T+MMF	Bloating × 3 weeks	C/A/T	Y	Y	N	2.2	90	23	XR × 1	Flagyl O_2_ at 15 L NPO × 1 day
10	37, F	Scleroderma ILD	28	38	P+T+MMF	None	C/A/T/D	Y	Y	N	0.9		24	XR × 3	O_2_ at 15 L NPO × 1 d
11	79, M	IPF	375		P+T+MMF	None	C/A	Y	N	N	2	39	23	CT × 2 XR × 1	O_2_ at 15 L NPO × 1 d
12	54, F	IPF	28	37	P+T+MMF	None	C/A/T/D	Y	Y	N	1.6		26	XR × 1	Flagyl O_2_ at 15 L Switched TF to Vivonex
13	71, M	IPF	209	10	P+T+MMF	N/V/D × 10 d	C	N	N	Y	1.6		28	XR × 5	NPO × 1 d Flagyl O_2_ at 15 L
14	61, F	Scleroderma ILD	47	53	P+T+MMF	None	C/A/T	Y	Y	N	2.6		24	XR × 3	NPO × 1 d O_2_ at 15 L Zosyn
15	73, M	CPFE	37	45	P+T+MMF	Diarrhea	C/A/T	Y	Y	N	3.3	74	28	CT × 2 XR × 4	NPO × 3 days O_2_ at 15 L Flagyl/Vancomycin (C. diff)
16	61, M	IPF	63	21	P+T+MMF	Diarrhea	C/A/T/D	Y	Y	N	1.8	38	25	XR × 4	Switch TF to elemental Flagyl, Florastor O_2_ at 15 L
17	59, F	IPF	234	193	P+T+MMF	None	C/A/T/D	N	N	N	0.5		24	None	Patient seen in ED after outside X-ray showing PI, discharged from ED
18	43, M	IPF	200	108	P+T+MMF	None	A/T	Y	Y	N	1.8	34	25	XR × 7	Switch TF to elemental O_2_ at 15 L Flagyl
19	71, M	IPF	24	13	P+T+MMF	None	C	Y	N	N	1.5		26	None	Treated outpatient
20	58, M	A1AT	17	28	P+T+MMF	Diarrhea	C/A/T/D	Y	Y	N	0.6	120	30	XR × 2	NPO × 1 day Flagyl O_2_ at 15 L
21	55, M	IPF	1477	30	P+T	None	C/A	N	Y	N	1.5	23	36	XR × 3	NPO × 1 day O_2_ at 15 L
22	69, M	IPF	51	39	P+T+MMF	None	C/A/T	Y	Y	N	0.8	40	29	None	NPO × 1 day O_2_ at 15 L
23	62, M	IPF	202		P+T+MMF	Emesis × 1	C/A/T	N	N	N	1		22	XR × 3	NPO × 1 day O_2_ at 15 L

Note: IPF: interstitial pulmonary fibrosis; P: prednisone; T: tacrolimus; MMF: mycophenolate mofetil; C/A/T/D: cecum/ascending/transverse/descending colon.

**Table 2:** Thompson et al. patients.

Patient	Age, sex	Primary diagnosis	Time to PI after Tx	Immunosuppression	Symptoms	PI distribution	Pneumoperitoneum	Lactic acid
1	39, F	Scleroderma ILD	84	P+T+A	Abdominal pain, N/V	C/A/T/D	N	1.3
2	64, M	IPF	453	P+T	Abdominal pain	C/A	Y	1.1
3	51, M	Cystic fibrosis	128	P+T+A	None	C/A/T/D	Y	1
4	55, M	COPD	24	P+T+A	Diarrhea	C/A/T	Y	1.7
5	65, M	IPF	125	P+T+A	None	C/A/T/D	N	1.3
6	22, M	Cystic fibrosis	16	P+T+A	Abdominal pain	C/A	N	1
			83					0.7

Note: A: azathioprine.

**Table 3:** Chandola et al. data.

Patient	Age, sex	Primary diagnosis	Time to PI after Tx	Immunosuppression	Symptoms	PI distribution	Pneumoperitoneum	Portomesenteric gas	Lactic acid
1	56, M	COPD	73	P+T+MM+B	Diarrhea, vomit	A/T/D	N	N	
2	59, M	COPD	160	P+T+MM+B	Abdominal pain	C/A/T/D	Y	N	4.1
3	32, F	GVHD	134	P+T+MMF+A+C	Diarrhea	A/T	Y	N	1.1
4	59, M	IPF	19	P+T+MM	Diarrhea	A/T	Y	N	1.2
5	35, F	IPF	63	P+T+MM+B	Abdominal pain	Terminal ileum	N	N	1.7
6	53, F	COPD	5	P+T+MM+B	Distention	A	N	Y	10.5
7	68, M	IPF	90	P+T+MM+B	None	A/T	N	N	
8	63, F	IPF	455	P+T+MM+B	None	T	Y	N	1.8
9	42, F	Sarcoidosis	2495	P+T+MM	None	C/A/T	N	N	
10	49, M	COPD	34	P+T+MM	None	A/T	Y	N	
